# Supplementary material for: Splice-Junction-Based Mapping of Alternative Isoforms in the Human Proteome
Source: Cell Rep. Author manuscript; Available in PMC 2020 Jan 15. (PMC6961840; doi:10.1016/j.celrep.2019.11.026)
Supplement: 2 [file NIHMS1546469-supplement-2.zip › DF1/PXD010154/Liver/Liver_33_FRMD4A_HCILLPGSALGLLMMTEGR.pdf]

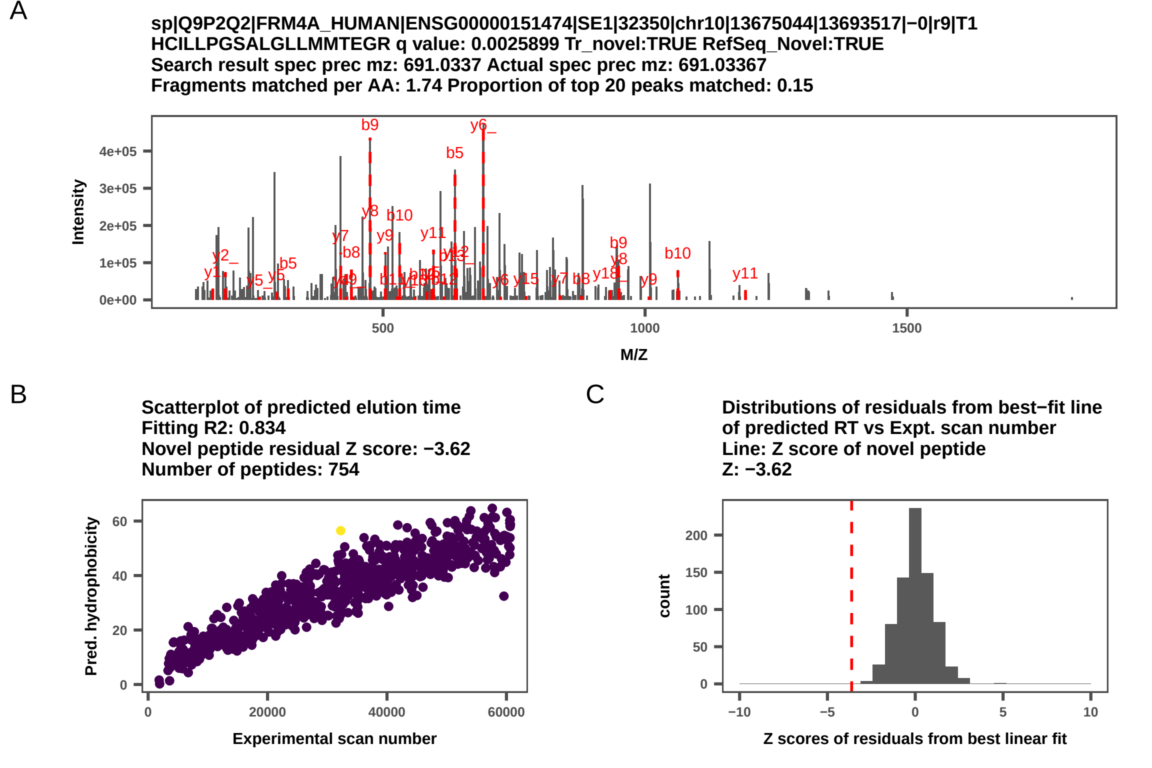

A
sp|Q9P2Q2|FRM4A_HUMAN|ENSG00000151474|SE1|32350|chr10|13675044|13693517|−0|r9|T1
HCILLPGSALGLLMMTEGR q value: 0.0025899 Tr_novel:TRUE RefSeq_Novel:TRUE
Search result spec prec mz: 691.0337 Actual spec prec mz: 691.03367
Fragments matched per AA: 1.74 Proportion of top 20 peaks matched: 0.15
Intensity
4e+05
3e+05
2e+05
1e+05
0e+00
b9
y6_
b5
y8
b10
y7
y9
y11
b8
y2_
b5
y1
y5_
y5
y6
y15
y7
b8
y18
b9
y8
b10
y9
y11
500
1000
1500
M/Z
B
Scatterplot of predicted elution time
Fitting R2: 0.834
Novel peptide residual Z score: −3.62
Number of peptides: 754
Pred. hydrophobicity
60
40
20
0
0
20000
40000
60000
Experimental scan number
C
Distributions of residuals from best−fit line
of predicted RT vs Expt. scan number
Line: Z score of novel peptide
Z: −3.62
count
200
150
100
50
0
−10
−5
0
5
10
Z scores of residuals from best linear fit
